# A non-randomised, single-centre comparison of induction chemotherapy followed by radiochemotherapy versus concomitant chemotherapy with hyperfractionated radiotherapy in inoperable head and neck carcinomas

**DOI:** 10.1186/1471-2407-6-30

**Published:** 2006-02-01

**Authors:** Reinhold Graf, Bert Hildebrandt, Wolfgang Tilly, Hanno Riess, Roland Felix, Volker Budach, Peter Wust

**Affiliations:** 1 Clinic for Radiation Medicine, Charité Medical School, Campus Virchow-Klinikum, Augustenburger Platz,113353 Berlin, Germany; 2 Medical Clinic for Hematology and Oncology, Charité Medical School, Campus Virchow-Klinikum, Augustenburger Platz 1, 13353 Berlin, Germany; 3 Clinic for Radiation Oncology, Charité Medical School, Campus Charité Mitte, Schumannstr. 21, 10117 Berlin, Germany

## Abstract

**Background:**

The application of induction chemotherapy failed to provide a consistent benefit for local control in primary treatment of advanced head and neck (H&N) cancers. The aim of this study was to compare the results of concomitant application of radiochemotherapy for treating locally advanced head-and-neck carcinoma in comparison with the former standard of sequential radiochemotherapy.

**Methods:**

Between 1987 and 1995 we treated 122 patients with unresectable (stage IV head and neck) cancer by two different protocols. The *sequential protocol *(SEQ; 1987–1992) started with two courses of neoadjuvant chemotherapy (cisplatin [CDDP] + 120-h continuous infusions (c.i.) of folinic acid [FA] and 5-fluorouracil [5-FU]), followed by a course of radiochemotherapy using conventional fractionation up to 70 Gy. The *concomitant protocol *(CON; since 1993) combined two courses of FA/5-FU c.i. plus mitomycin (MMC) concomitantly with a course of radiotherapy up to 30 Gy in conventional fractionation, followed by a hyperfractionated course up to 72 Gy. Results from the two groups were compared.

**Results:**

Patient and tumor characteristics were balanced (SEQ = 70, CON = 52 pts.). Mean radiation dose achieved (65.3 Gy vs. 71.6 Gy, p = 0.00), response rates (67 vs. 90 % for primary, p = 0.02), and local control (LC; 17.6% vs. 41%, p = 0.03), were significantly lower in the SEQ group, revealing a trend towards lower disease-specific (DSS; 19.8% vs. 31.4%, p = 0.08) and overall (14.7% vs. 23.7%, p = 0.11) survival rates after 5 years. Mucositis grades III and IV prevailed in the CON group (54% versus 44%). Late toxicity was similar in both groups.

**Conclusion:**

Concurrent chemotherapy seemed more effective in treating head and neck tumors than induction chemotherapy followed by chemoradiation, resulting in better local control and a trend towards improved survival.

## Background

Various approaches have been developed to intensify standard radiotherapy in locally advanced head and neck carcinomas, such as altered fractionation schemes (hyperfractionation, acceleration) [1] or combination with chemotherapy [2]. Improved local control and survival have been confirmed in recent trials of concomitant radiochemotherapy [2-4]. However, three meta-analyses of radiochemotherapy in advanced head and neck cancer [5] showed that the impact of chemotherapy on overall survival is rather small (improving e.g. 5-year overall survival from 30% to 35%). Compared to radiotherapy alone, concomitant radiochemotherapy shows the biggest improvement with respect to local control and disease-specific survival. As a result, concomitant radiochemotherapy with cisplatin and 5-FU is increasingly accepted as standard treatment for patients with unresectable head and neck carcinoma and adequate performance status.

To date, no randomised study has compared induction chemotherapy followed by concurrent radiotherapy against chemotherapy with a concomitant radiochemotherapy schedule. We present such a non-randomised comparison with respect to late toxicity, local control and survival in patients with stage IV unresectable head and neck cancers, who were treated according to two different schedules at our institution between 1987 and 1995.

## Methods

Between March 1987 and July 1995, 122 patients were treated with two consecutive phase-II protocols in the departments of radiation oncology and hematology/oncology at the Charité. The first, sequential (SEQ) protocol was performed between 1987 and 1992 and included neoadjuvant chemotherapy with a subsequent radiotherapy course. The second protocol was performed after 1992 and consisted of concomitant radiochemotherapy with subsequent hyperfractionation.

The first protocol was approved by the ethical committee at the Virchow-Klinikum and the second by the ethical committee of the Campus Charité-Mitte of the Charité.

### Eligibility criteria

Patients were eligible if they had primary, histologically proven, non-metastatic squamous cell carcinoma of the oral cavity, oropharynx, hypopharynx or larynx, assessed as unresectable in the department of head-and-neck surgery because of size, extension or location. Patients who had previously undergone cytostatic therapy or radiotherapy were excluded, along with those with contraindications against radiotherapy or the cytostatic agents used (5-FU, FA, CDDP, MMC). Written informed consent was obtained from all patients before therapy.

### Evaluation of patients

For every patient, clinical, laboratory and imaging examinations were performed to achieve an accurate TNM stage (UICC, 5^th ^ed.) [6] and to allow follow-up.

During therapy, the patients were examined for acute toxicity every week. Acute side effects were indexed according to EORTC criteria [7]; late side effects were classified according to the LENT tables [8]. The response (primary as well as lymph nodes) was assessed by clinical examination and CT according to WHO criteria [9].

After completion of therapy, each patient was followed up within 6–8 weeks by an interdisciplinary consultation involving clinical evaluation (head and neck status) complemented by CT. After specific symptoms or suspicious findings, diagnostic procedures were extended. Further follow-up included examinations at intervals extending from 6 weeks initially to 6 – 12 months after the third year.

In cases of histologically proven local recurrence, all available salvage treatment options were offered (brachytherapy, hyperthermia, external re-irradiation, chemotherapy); most often palliative chemotherapy with MTX (methotrexate) was performed.

### Treatment records (fig. 1A+B)

In the first sequential (SEQ) protocol, 2 three-weekly courses of neoadjuvant chemotherapy were applied, consisting of cisplatin (CDDP; 100 mg/m^2 ^i.v., on day 1) followed by 50 mg/m^2 ^folinic acid (day 1) and parallel 120 h continuous infusions of folinic acid (250 mg/m^2 ^per day, days 1–5) and 5-fluorouracil (600 mg/m^2^, days 1–5). Six weeks later (day 43), the third chemotherapy course was started at a 60% reduced dose concomitantly with the definitive radiotherapy. External beam radiotherapy was applied without preference to both groups, with photon energies of 1.2 MeV (telecobalt unit) or 4–8 MV (linear accelerator). The primary tumor and regional lymph drainage areas received 60 Gy in single fractions of 1.8–2 Gy. To shield the spinal cord after achieving a dose of 40 Gy, the dorsal parts of the target volume were treated with fast electrons of 9-12MeV. Primary tumor and lymph node areas that were macroscopically involved were boosted to a total dose of 70 Gy.

In the second, concomitant (CON) schedule, an RCT protocol was conducted concomitantly with chemotherapy as in the study of Budach [4,10]. Here, chemotherapy was initiated with intravenous short infusions of folinic acid (50 mg/m^2^, day 1) and 5-FU (350 mg/m^2^, day 1), followed by parallel 120 h continuous infusions with both drugs (folinic acid, 100 mg/m^2 ^daily; 5-FU, 350 mg/m^2 ^daily). In addition, short infusions of mitomycin C (10 mg/m^2^) were administered on days 1 and 36. Radiotherapy began synchronously with chemotherapy, starting with single doses of 2 Gy up to a total dose of 30 Gy over the entire affected volume. Gross areas were then boosted using hyperfractionation, with two daily 1.4 Gy fractions separated by an interval of at least 6 hours, to a total dose of 70.6 Gy (10 patients) or 72 Gy (42 patients). Lymph node regions at low or intermediate risk of subclinical infiltration were irradiated to total doses of 50 Gy (normofractionated) or 60 Gy (hyperfractionated after 30 Gy), respectively. The dorsal paravertebral parts of the target volume were irradiated with lateral 6–9 MeV electron fields after 36–40 Gy at the myelon, conventionally fractionated if not clinically involved, hyperfractionated otherwise.

### Statistical evaluation

We used the distribution-free Mann-Whitney test to compare the two patient groups (SEQ/CON). A univariate analysis of prognostic factors was carried out through a comparison of survival times and local control rates using the log-rank test. A multivariate analysis was carried out for all parameters with significant correlations in the univariate analysis according to the proportional-hazard model. Time-to-progression (TTP), tumour specific and overall survival were identified from the first day of treatment up to the day of death or last patient contact following the Kaplan-Meier method [11]. In particular, TTP was defined as the time from day 1 of treatment until clinical progression, i.e. the timing of a more than 25% increase in post-therapeutic volume (determined 6–8 weeks after end of treatment) in any tumor lesion, or histopathological validation of recurrent disease after a CR.

## Results

Seventy patients in the SEQ group and 52 patients in the CON group were included in an intent-to-treat analysis. The median observation times for the surviving patients were 53 months for the SEQ group and 41 for the CON group, respectively (for all patients, 19.8 and 14.7 months in the two groups).

### Patients' characteristics

We found a comparable distribution of prognostic factors in both groups with regard to age, gender and performance status (WHO). Tumors of the oropharynx predominated, with frequencies of 54% and 58% in the SEQ and CON group, respectively (p = 0.66). The percentage of tumors located in the hypopharynx was found to be slightly higher in the SEQ group (29% vs. 17% of patients, p = 0.13). All other tumor characteristics were statistically equally distributed according to table 1. Most patients had advanced stages, with T4-categories in 68% vs. 77% (p = 0.28), and N2c-N3 in 44% vs. 43%, of patients in the SEQ vs. CON groups, respectively. The resulting clinical stages were as high as IV and V (RTOG) for the majority of the patients, i.e. 33% and 59% (together 92%) for the SEQ group and 31% and 67% (together 98%, p = 0.37) for the CON group.

### Treatment parameters

In the SEQ group, 5/70 patients (7%) received only one chemotherapy course owing to rapid tumor progression and/or poor performance status. Two courses were applied to 22/70 patients (31%), and three cycles were given to only 43/70 (62%). Of these, 65/70 (95%) could be evaluated for response after induction chemotherapy, and 62/70 (89%) after the whole treatment course. Patients who could not be evaluated for response included three treatment-related deaths during the first two cycles. At the second checkpoint (after radiotherapy), 4/70 patients were non-evaluable and received no radiotherapy.

In the CON group, the first course of chemotherapy was given to all patients with at least 80% of the prescribed 5-FU dose level. Two of the patients received only one application of mitomycin C in week 1 because the whole treatment had resulted in considerable toxicity by week 6. Only in 1/52 of the patients (2%) was radiochemotherapy abandoned, so 51/52, i.e. 98%, of the patients were further evaluable.

On average, we applied an average radiation dose of 65.3 Gy to the primary tumor in the SEQ group and 71.6 Gy in the CON group. The corresponding mean radiation doses at the involved lymph nodes were 58.1 Gy and 69.5 Gy, respectively. These doses were significantly different in the two groups (p = 0.00).

In 23/61 (36%) of the patients in the SEQ group, radiotherapy was interrupted for longer than 1 week, while 14/61 (23%) had a break for longer than 3 weeks. In contrast, only 7/51 (14%) of the patients in the CON group had a break of at least 1 week, and 3/51 (6%) for more than 2 weeks (p = 0.00). In consequence, the average duration of radiotherapy (if performed) was significantly longer in the SEQ compared with the CON group (79.9 vs. 43.4 days, p = 0.00).

### Toxicity (table 2)

Three treatment-related deaths occurred in the SEQ-group (due to hematotoxicity), whereas no such serious complication was found in patients assigned to the CON group. Acute local toxicity was generally moderate in both groups, except for severe mucositis, which affected 54% of patients receiving hyperfractionated radiotherapy in the CON-group compared with 44% in SEQ group (n.s.). Regarding late toxicity, lymph edema, mouth dryness and dysphagia were the most frequently observed grade III and IV side effects in both groups, as summarized in table 2. The frequency of grade I or II acute toxicity was higher, as expected, in the CON group. Occurrences of grade I or II late toxicity proved to be equally distributed.

### Response

The complete primary tumor remission (CR) rates were 41% in the SEQ vs. 46% in the CON group, and the partial remission (PR) rates were 26% vs. 44%, respectively. Response in the lymph nodes was also less frequent in the SEQ than the CON group (86% vs. 98%). A survey of the response rates in primary tumors and involved lymph nodes is given in table 3. The overall differences (CR + PR + PD/NC) in response (CON vs. SEQ) were significant for both the primary tumors (p = 0.02) and the lymph nodes (p = 0.00), but the single endpoints (CR or PR or PD/NC) did not show significant between-group differences. Calculations were performed on the total number of eligible patients, but it is notable that the proportion of non-evaluable patients was considerably higher in the SEQ group (8/70 versus 1/52) and included four patients receiving no radiotherapy at all (three were treatment-related deaths). This difference in treatment failure and disruption is a further criterion in favour of the CON group, but it is not encompassed by the response analysis.

### Local control and survival

**Figure 2:**
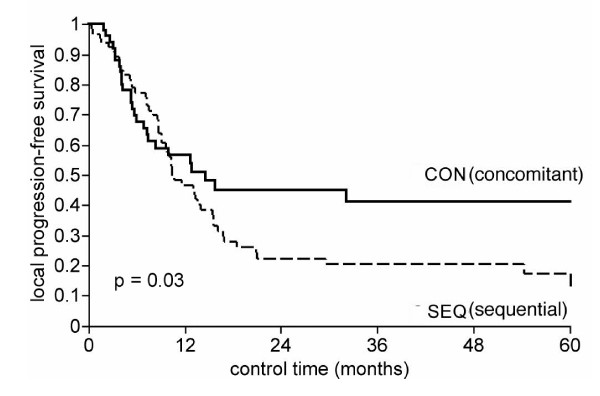
Kaplan-Meier survival curves with locoregional control, i.e. detecting time-to-local progression, in relation to the type of protocol.

Calculations were performed on the eligible patients and the results are summarised in table 4. The 5-year local control rate (local-progression-free survival) was significantly lower in the SEQ-group than in the CON-group according to fig. 2 (17.6% vs 41%, p = 0.03 with 51 patients vs. 26 patients relapsing locally). Among the patients achieving complete remission, 29% had recurrences of the primary and 24% in the lymph nodes in the CON group after 5 years, compared with 55% and 48% in the SEQ group. The most common site of recurrence in the SEQ-group was locoregional (90% of progressive patients), whereas distant metastases (DM) alone or combined with local failure (LF) occurred very rarely (4% or 6%, DM ± LF). In contrast, only 73% had LF alone in the CON-group, whereas 17% of the patients suffered from initial DM (± LF). Overall, fewer patients in the SEQ-group than in the CON-group presented with DM (± LF) (actuarially 18% or 26% after 5 years, 6 patients vs. 10 patients). Secondary cancers occurred in both groups with similar frequencies (7% and 8%).

**Figure 3:**
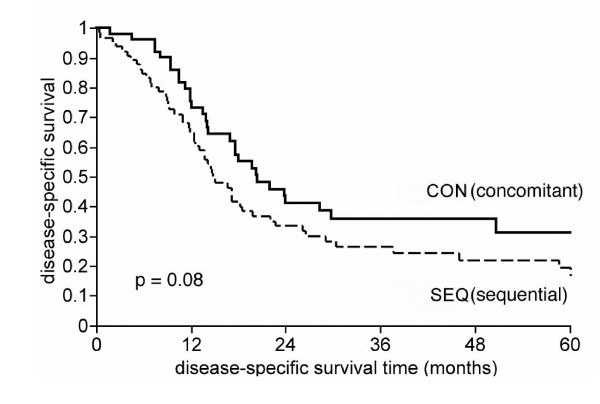
Kaplan-Meier disease-specific survival curves, i.e. considering tumor-related deaths, in relation to the type of protocol.

**Figure 4:**
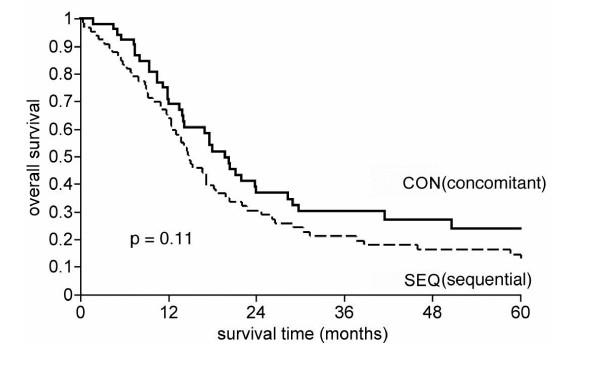
Kaplan-Meier overall survival curves, i.e. considering all causes of death, in relation to the type of protocol.

The actuarial disease-specific survival curves (fig. 3, table 4) were not statistically different (5 years: 19.8% and 31.4%, with tumor-related deaths of 52 patients and 30 patients), but showed a trend in favour of the CON-group (p = 0.08). The overall survival curves showed the same tendency, with 3-year survival rates of 30% vs. 21% and 5-year survival rates of 24% vs. 15% in favour of the CON-group (p = 0.11, 56 vs. 36 patients dying from all causes) (fig. 4).

### Predictors of outcome

The prognostic factors that had proved significant in univariate analysis were subjected to multivariate analysis, which showed that T-categories (≤ T3 vs. T4, p < 0.001) and N-categories (≤ N2a vs. N2b, N3, p < 0.01) were significantly correlated with locoregional control. The type of treatment protocol had a borderline significant relationship (p = 0.06) with local treatment outcome. Performance status, RTOG stage and radiation dose proved not to be significantly correlated in the multivariate analysis.

With regard to disease-specific survival, a multivariate analysis of the significant prognostic factors from the univariate analysis revealed T-stage (≤ T3 vs. T4, p < 0.001), N-stage (≤ N2a vs. N2b, N3, p < 0.001), performance status (p = 0.04) and treatment protocol (SEQ vs. CON, p < 0.01) as significant predictors. The total dose showed borderline significant correlation (p = 0.06) with disease-specific survival, whereas there was no significant correlation for the RTOG stage. N-stage (p = 0.00), treatment protocol (p = 0.03) and radiation dose (p = 0.05) proved significant for overall survival.

## Discussion

Radiochemotherapy is becoming increasingly established as a standard method for treating inoperable head and neck carcinomas, but the optimal scheme is still under discussion. One reason is the large variation in risk factors among patient groups.

It has been found that the percentage of local control after 5 years following a treatment regimen consisting solely of radiotherapy is improved by adding chemotherapy (5-FU, cisplatin etc.), which has conferred survival benefit in a number of trials. Several studies have failed to demonstrate any influence on the frequency of systemic dissemination.

We evaluated two different radiochemotherapeutic approaches to patients with locally advanced head and neck cancer. Prognostic factors were more or less equally distributed in the two groups. Clearly, the groups differed in treatment parameters in concerning radiation dose, cytotoxic drugs, treatment time, etc. Therefore, it might be difficult to estimate the influence of a single parameter on treatment outcome. In our study, improvements in disease staging and support measures could not have been major contributors. Treatment technique, treatment planning and radiation therapy technology were not changed significantly in our institution during the time encompassing the studies on the two groups.

In general, our results suggest that concomitant radiochemotherapy is superior with respect to local control, but not unequivocally with respect to distant metastases or overall survival. The regenerative response of clonogens may weaken the contribution of induction chemotherapy to locoregional control. In addition, the toxicity of the applied dose-intense chemotherapy often impaired the regular administration of radiotherapy within the SEQ-group (an attribute of the sequential scheme), resulting in treatment interruptions that led to a mean treatment time twice as long as in the CON-group. This shows that the impairment in quality of life following sequential treatment – in addition to worse results – may be greater than the adverse effects of concomitant radiochemotherapy.

The 5-year overall survival rates (15% vs. 24%) and 5-year disease-free survival rates (20% vs. 31%) are relatively low in both groups, obviously because all except one patient suffered from stage IV disease, and a high proportion of patients in both groups had *unfavourable prognostic indicators *(see table 1). Other investigators report similarly low overall survival rates for patients with comparably unfavourable prognostic factors [4,12,13].

Better treatment results are achieved with *selected *collectives. Regarding recent prospective trials, 3-year local control rates of 50–70% are not uncommon in patients treated with irradiation and concurrent chemotherapy (with conventional or altered fractionation) [14]. For example, Dinges et al. [10] reported a high disease-free survival rate of 59% and a local control rate of 72% after 4 years using the treatment schedule applied in the CON-group of our analysis. Overall, most of the published studies [14] report 2–3 year overall survival rates of around 20–30% following radiotherapy and 40–50% following concomitant radiochemotherapy [3,14-16], comparable to our 30–40% 2-3-year survival rate after concomitant RCT (fig. 5). The difference of about 10% can easily be explained by the poor prognostic indicators of our (rather unselected) patient groups. Note that in a large meta-analysis with more than 10,000 patients, the survival rates for any kind of locoregional treatment plus chemotherapy (including radical surgery with adjuvant or neoadjuvant chemotherapy) were not higher than 36% after 5 years [5], compared to 24% in our CON group.

Patients with unresectable head and neck tumors have a limited risk of death from distant metastases, at least in comparison to patients suffering from gastrointestinal malignancies. Studies based on autopsies have verified that 40% of patients with head and neck cancers died from local relapses without evidence of distant metastases or secondary primary tumors (32%), whereas only 5% of patients died from distant metastases alone [17].

Theoretically, neoadjuvant chemotherapy can benefit a few patients directly (by tumor regression in responders), but may also reduce the incidence of distant metastases, especially in collectives with known risk factors. In a number of clinical studies of induction chemotherapy [18], neither significant downstaging nor a decreased incidence of distant metastases was observed. In a meta-analysis, Pignon found no significant benefit from induction chemotherapy as opposed to concomitant chemotherapy in head and neck cancer [5]. In our study, local failures were the most frequent causes of death, and so far as local control is concerned we found a markedly better outcome in the CON-group. On the other hand, patients with metachronous distant metastases were less frequent in the SEQ-group.

Randomised studies comparing induction chemotherapy with subsequent radiotherapy against radiotherapy alone [2,15] have failed to showany significant advantage in terms of overall survival and disease-free survival. On the other hand, concomitant application of cytostatic drugs has resulted in significant improvement, as shown in several randomised trials, independently of fractionation [2,3,19]. Radiochemotherapy can even improve on radiotherapy alone with dose escalation [4]. In other studies, a randomised comparison between hyperfractionated dose-escalated radiotherapy and hyperfractionated radiotherapy with concomitant chemotherapy showed only slight advantages for the combined arm [20]. Only a few studies have been conducted on randomised comparisons between concomitant radiochemotherapy (with moderate chemotherapy dosages) and sequential radiochemotherapy (with systemically effective chemotherapy dosages). In this regard, Adelstein [2] reported a significant increase in the local control rate using concomitant RCT. Taylor [21] achieved significantly better disease-free survival, although overall survival was not significantly increased.

## Conclusion

In comparison with concomitant radiochemotherapy, induction chemotherapy followed by radiotherapy has lost popularity. This was confirmed by our retrospective analysis, i.e. treatment with induction chemotherapy proved inferior with regard to applicability and efficacy and resulted in unsatisfactory survival rates and low patient compliance. Although induction chemotherapy might result in higher systemic effectiveness, inferior locoregional control dominates the clinical outcome. Schemes using simultaneous chemotherapy and hyperfractionated radiotherapy generally show greater acute toxicity and better overall survival, tumour specific survival, disease-free survival and local control if patient selection is appropriate. To optimise the combination of both modalities (radiotherapy and chemotherapy) the aim must be to achieve higher local effectiveness together with tolerable acute and late toxicities.

## Abbreviations

Head-and-neck carcinoma (H&N); sequential protocol (SEQ); cisplatin (CDDP); folinic acid (FA); 5-fluorouracil (5-FU); concomitant protocol (CON); mitomycin (MMC); local control (LC); disease-specific survival (DSS); MTX (methotrexate); time-to-progression (TTP); complete remission (CR); partial remission (PR); distant metastases (DM); local failure (LF).

## Competing interests

The author(s) declare that they have no competing interests.

## Authors' contributions

R.G. performed the radiotherapy, analysed and interpreted the data, and drafted the tables, figures and manuscript. B. H. performed the chemotherapy and critically revised the manuscript. W. T. performed parts of the statistical analysis and contributed to the figures. H. R. was the consultant for chemotherapy and established induction chemotherapy plus RCT. R. F. performed the follow-up and was the consultant for diagnostics. V. B. established the concomitant RCT and was the consultant for radiotherapy. P. W. supervised the acquisition, analysis and interpretation of data, and conceived and revised the manuscript.

## Pre-publication history

The pre-publication history for this paper can be accessed here:



## Figures and Tables

**Figure 1:**
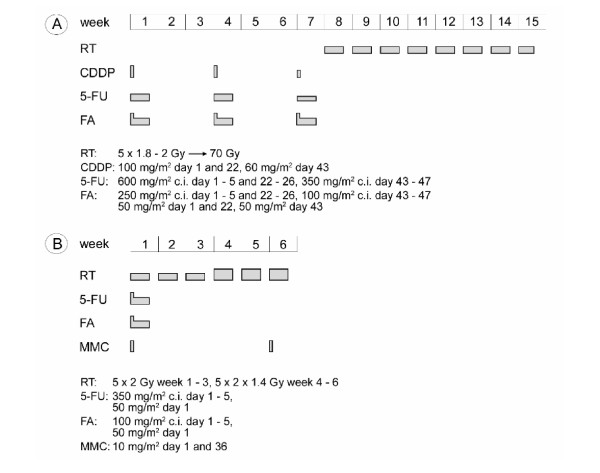
Flow sheet of the two treatment regimes. A (top): treatment schema, sequential protocol (SEQ.). B (bottom): treatment schema, concomitant protocol (CON).

**Table 1:** Comparison of patients' characteristics differentiated to the type of schedule

	**SEQ (sequential, n = 70)**		**CON (concomitant, n = 52)**		**p**
Parameter	number	(%)	number	(%)	0.80

Age, years					
Mean (Range)	53 (37–77)		52.8 (39–66)		0.79
Performance status (WHO)					0.67
0,1	35	(50)	30	(58)	
2,3	27	(39)	19	(37)	
Unknown	8	(11)	2	(4)	
T-stage (UICC)					0.54
T2	4	(6)	3	(6)	
T3	18	(26)	9	(17)	
T4	48	(68)	40	(77)	
N-stage					0.13
N2c	23	(33)	15	(29)	
N3	8	(11)	7	(14)	
RTOG stage groupings					0.58
III (T3-4 N0, T1-2 N2)	5	(7)	1	(2)	
IV (T1-2 N3, T3 N1-2, T4 N1)	23	(33)	16	(31)	
V (T4 N2-3)	41	(59)	35	(67)	
UICC stage groupings					
IV (a + b)	69	(99)	52	(100)	
Tumor location					0.19
Oral cavity	7	(10)	10	(19)	
Oropharynx	38	(54)	30	(58)	
Hypopharynx, Larynx	25	(36)	12	(23)	
Mean tumor size (cm)	4.8		4.6		0.46

**Table 2:** Acute and late toxicity after radiotherapy and chemotherapy according to RTOG/ECOG and LENT scores (highest level given)

	**SEQ**				**CON**				
	**Grade III**		**Grade IV**		**Grade III**		**Grade IV**		**p**

	**n**	**(%)**	**n**	**(%)**	**n**	**(%)**	**N**	**(%)**	

**Acute toxicity (RTOG)**									
Skin	11	(16)	0		5	(10)	0		n.s.
Mucositis	31	(44)	1	(1)	28	(54)	2	(4)	n.s.
Pharynx	9	(13)	1	(1)	9	(17)	1	(2)	n.s.
Salivary glands	10	(14)	0		4	(8)	0		n.s.
**Late toxicity (LENT scores)**									
Skin	2	(3)	0		2	(4)	0		n.s.
Subcutis	5	(7)	0		7	(14)	0		n.s.
Mucosa	4	(6)	0		3	(6)	0		n.s.
Salivary glands	7	(10)	0		3	(6)	0		n.s.
Pharynx	5	(7)	0		4	(8)	1	(2)	n.s.
Larynx	3	(4)	0		2	(4)	0		n.s.
Mandibula	1	(1)	0		0		1	(2)	n.s.

**Table 3:** Comparison of responses in dependency on the schedule

	SEQ (n = 70)				CON (n = 52)			
	After 2 cycles chemotherapy		After chemotherapy and radiotherapy		After chemotherapy and radiotherapy			

	Primary (%)	Nodes (%)	Primary (%)	Nodes (%)	Primary (%)	p^2)^	Nodes (%)	p^2)^

Evaluable	93 (65/70)	93 (65/70)	89 (62/70)	86 (60/70)	98 (51/52)	0.02	98 (51/52)	0.00
Complete response ^1) ^(CR)	11	7	41	33	46	n.s.	58	n.s.
Partial remission ^1) ^(PR)	51	51	26	33	44	n.s.	37	n.s.
CR and PR	63	59	67	66	90	n.s.	94	n.s.
NC, Progressive disease	30	33	21	20	8	n.s.	4	n.s.

^1) ^*WHO *

^2) ^*compared with response in the SEQ-group after chemotherapy and radiotherapy*

**Table 4:** Comparison of treatment results between the two groups

	**SEQ**	**CON**	**p**
Overall survival (5 ys.)	14.7 %	23.7 %	0.11
Median overall survival	14.6 months	20.1 months	
Disease-free survival (5 ys.)	19.8 %	31.4 %	0.08
Progression free survival (5 ys.) years)	17.6 %	41.0%	0.03
Median time to (local) progression	10.7 months	15.0 months	

^1) ^*WHO *^2) ^*compared with response in the SEQ-group after chemotherapy and radiotherapy*
